# The relationship between climate change and malaria in South-East Asia: A systematic review of the evidence

**DOI:** 10.12688/f1000research.125294.2

**Published:** 2023-07-21

**Authors:** Ardhi Arsala Rahmani, Dewi Susanna, Tommi Febrian

**Affiliations:** 1 Doctoral Program in Public Health, Universitas Indonesia, Depok, Jawa Barat, 16424, Indonesia; 2 Department of Environmental Health, Faculty of Public Health, Universitas Indonesia, Depok, Jawa Barat, 16424, Indonesia; 3 Global Green Growth Institute (GGGI), Jakarta, Daerah Khusus Ibukota (DKI), 12950, Indonesia

**Keywords:** climate change, malaria, Southeast Asia, temperature, precipitation, windspeed, humidity, systematic review

## Abstract

**Background**: Climatic change is an inescapable fact that implies alterations in seasons where weather occurrences have their schedules shift from the regular and magnitudes intensify to more extreme variations over a multi-year period. Southeast Asia is one of the many regions experiencing changes in climate and concurrently still has endemicities of malaria. Given that previous studies have suggested the influence of climate on malaria’s vector the *Anopheles* mosquitoes and parasite the Plasmodium group, this study was conducted to review the evidence of associations made between malaria cases and climatic variables in Southeast Asia throughout a multi-year period.

**Methods**: Our systematic literature review was informed by the PRISMA guidelines and registered in PROSPERO: CRD42022301826 on 5 ^th^ February 2022. We searched for original articles in English and Indonesian that focused on the associations between climatic variables and malaria cases.

**Results**: The initial identification stage resulted in 535 records of possible relevance and after abstract screening and eligibility assessment we included 19 research articles for the systematic review. Based on the reviewed articles, changing temperatures, precipitation, humidity and windspeed were considered for statistical association across a multi-year period and are correlated with malaria cases in various regions throughout Southeast Asia.

**Conclusions**: According to the review of evidence, climatic variables that exhibited a statistically significant correlation with malaria cases include temperatures, precipitation, and humidity. The strength of each climatic variable varies across studies. Our systematic review of the limited evidence indicates that further research for the Southeast Asia region remains to be explored.

## Introduction

Climatic change, as an inescapable fact, refers to the changes in long-term normal weather conditions. Unlike the varying weathers due to seasonality that is of common occurrence in one given year, the climate is indicated by weather pattern types and related classifications such as the Köppen-Geiger, – which subdivides climates into the tropics, temperate, cold and polar. ^1^ The climate normal range is typically measured over a 30-year period but spans of 5- to 25-year periods have also recently been included. ^2^ A changing climate therefore also implies alterations in seasons where weather occurrences have their schedules shift from the regular and magnitudes intensify to more extreme variations over a multi-year period.

Naturally, the climate system has always changed across different geological epochs since our blue planet formed approximately 4.6 billion years ago. The epochs, which are part of a system of chronological dating, so called by geologists, paleontologists and paleoclimatogists, represent periods of geologic history. The current period, known as the Holocene, began around 11,000 years ago following the end of the Pleistocene epoch. The Holocene is marked by a relatively stable climate with averaging global temperature variations of about plus or minus 1°C for every turn of the century so far. ^3^ This marked stability has been advantageous for humanity and has allowed the establishment of modern civilizations, beginning with the advent of agriculture that relied on the very stable climatic conditions of the early Holocene. ^3^

An important feature of the current epoch is the natural greenhouse effect where the sun’s energy is partially absorbed by the earth’s surface and reflected back into space. The solar radiation is absorbed by naturally occurring greenhouse gas molecules which disperse the energy as heat thereby warming the lower atmosphere and conferring the needed energy and radiation to the biosphere. In the earth’s normal state, greenhouse gases, which include carbon dioxide (CO _2_), water vapour, nitrous oxides, and other compounds such as methane and ozone naturally exist around us. ^3^ However, with the ascent of industrialized civilization, humans have since caused atmospheric imbalances with increased accumulation of greenhouse gas emissions from anthropogenic activities. This has led to climatic variations and feedback, which include precipitation intensification as well as temperature rises. ^4^^,^^5^

These extreme feedbacks are of concern because they influence the reproduction of infectious agents such as viruses, bacteria and vectors such as mosquitoes and flies as they are sensitive to fluctuations in climatic variables such as temperature. ^6^ In the event of climatic fluctuations that enable reproductive enhancement of infectious agents, the spread of disease amongst humans inevitably increases and overall public health conditions are threatened. An example of this is malaria, wherein climatic fluctuations have been shown to influence the risk factors posed by both the infectious parasitic agent, the Plasmodium group, and *Anopheles* mosquitoes. ^7^^,^^8^ In fact, according to a study on malaria endemic Africa, optimum temperature window for malaria transmission is modelled to be in the 30-32°C range. The implication is that areas with a warming trend towards said range could potentially see persistent malaria endemicity. ^9^

Although malaria incidence and prevalence are also determined by variables like urbanization, globalization, migration patterns as well as land-use changes, ^10^ and the disease remains endemic in many parts of the world in spite of sociodemographic developments such as throughout Southeast Asia (WHO, 2020). In contrast to other malaria endemic regions such as sub-Saharan Africa, the particular attention to Southeast Asia is heeded on the fact that the region is home to a biodiverse array of malaria vectors ^11^ as well as dissimilar vector behaviors. ^12^

Therefore, with regards to the fact that malaria is a climate-dependent disease, it is endemic in many areas across Southeast Asia and the region having experienced climatic changes like the rest of the world, this study was conducted to critically assess and review the evidence of associations made between malaria cases and climatic variables in the region over a multi-year period, in line with the span of a climate normal range.

## Methods

### Search strategy

The conduct of this study was guided by the PRISMA 2020 checklist for review studies ^13^^,^^14^ and registered in PROSPERO: CRD42022301826 on 5 ^th^ February 2022. One Indonesian database (Garuda) and three international databases (PubMed, SpringerLink, ProQuest and Scopus) were searched to gather peer-reviewed articles for this review of evidence. The Indonesian database was included to capture additional articles catered in the authors’ local language as previously done in Babaie *et al*. (2018) ^15^ with the inclusion of Persian databases for a review of Iran and Fischer *et al*. (2020) ^16^ with the inclusion of German and French articles for a review of Europe. The search strategies applied were deployed in multiple sequences to mitigate any biases arising from missed articles from each database. The articles were then immediately disbursed amongst authors following selection. The keywords for our search included the terms ‘climate’, ‘ *iklim*’, ‘malaria’ and ‘Southeast Asia’ or the 10 Southeast Asian countries searched as ‘Indonesia’, ‘Malaysia’, ‘Singapore’, ‘Philippines’, ‘Thailand’, ‘Myanmar’, ‘Laos’, ‘Cambodia’, ‘Vietnam’ and ‘Brunei’. The search terms and methods along with the appropriate incorporation of truncations and operators specific to each database was discussed and consulted between DS and AR. Our search strategy did not include any limitations on publication periods. The search methods used and respective retrieved results from each database used are detailed as follows:

• PubMed (169 retrieved results): ((“climat*”[All Fields]) AND (“malaria”[All Fields]) AND (“Indonesia” OR “Malaysia” OR “Singapore” OR “Philippines” OR “Thailand” OR “Vietnam” OR “Laos” OR “Cambodia” OR “Myanmar” OR “Brunei” OR “Southeast Asia” [All Fields]))
• SpringerLink (126 retrieved results): where the the title contains “climate” AND “malaria” AND (“Indonesia” OR “Malaysia” OR “Singapore” OR “Philippines” OR “Thailand” OR “Vietnam” OR “Laos” OR “Cambodia” OR “Myanmar” OR “Brunei” OR “Southeast Asia”) with Content Type set to Articles
• Scopus (136 retrieved results): TITLE-ABS-KEY ((climat*) AND (malaria) AND (Indonesia) OR (Malaysia) OR (Singapore) OR (Philippines) OR (Thailand) OR (Vietnam) OR (Laos) OR (Cambodia) OR (Myanmar) OR (Brunei) OR (Southeast Asia)) AND (LIMIT-TO (DOCTYPE,“ar”))
• ProQuest (94 retrieved results): ALL (climat* AND malaria AND (Indonesia OR Malaysia OR Singapore OR Philippines OR Thailand OR Vietnam OR Laos OR Cambodia OR Myanmar OR Brunei OR Southeast Asia)) with Source Type set to Scholarly Journals
• Garuda (10 retrieved results): “iklim” AND “malaria”

### Inclusion and exclusion criteria

Original articles written in Indonesian and English which were analytical ecological studies and utilized longitudinal or time-series data of climatic variables with malaria incidence and/or prevalence in regions across Southeast Asia were included. Additionally, the articles included were studies which quantitatively analyzed the data through correlation, regression and/or mathematical models to infer relationships between multiple meteorological measures to reflect climate change and malaria incidence and/or prevalence. The studies that did not include data from a multi-year period and quantitative models with only one climatic variable were excluded.

### Study selection

**Figure 1.**
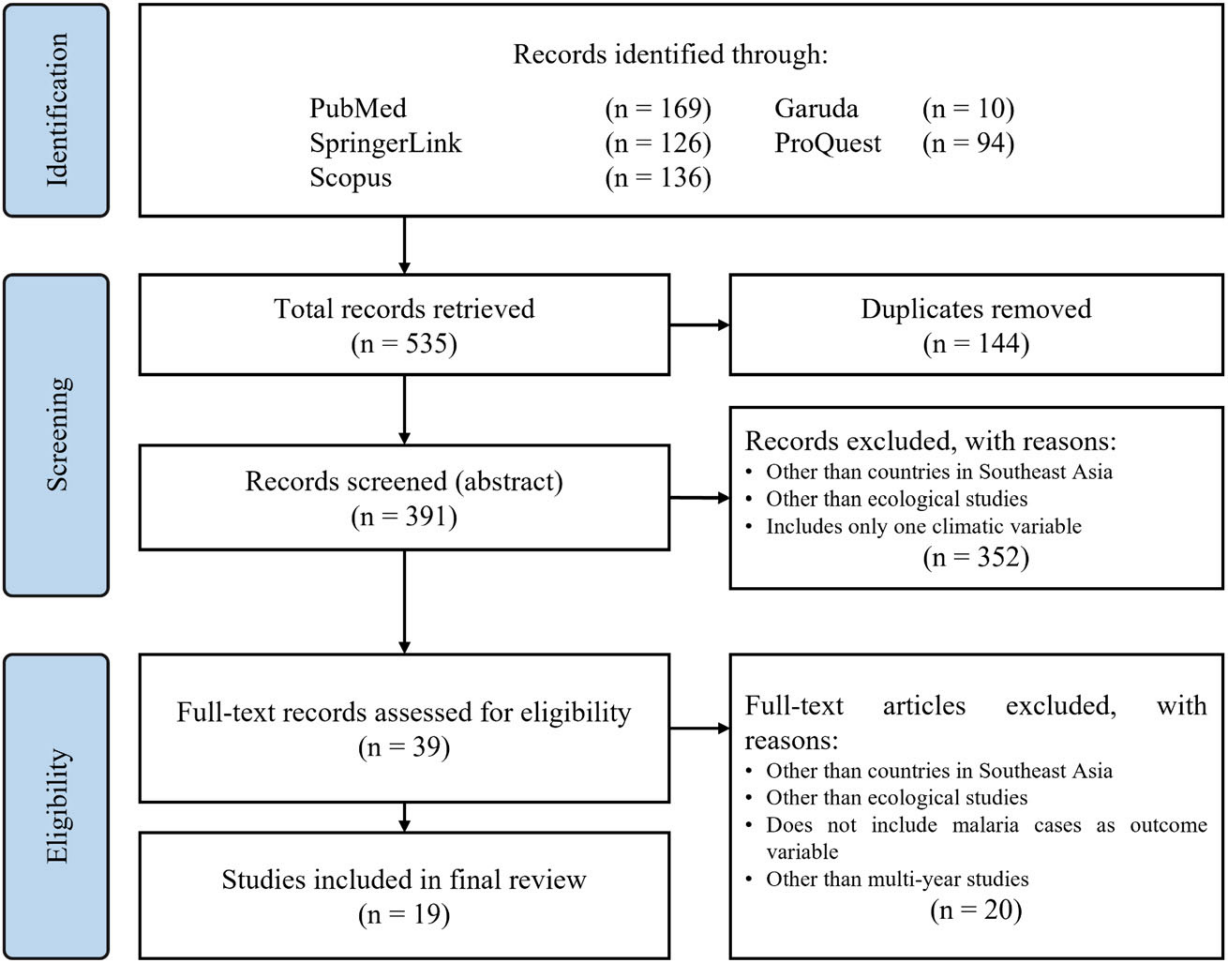
PRISMA review flowchart. ^45^

Three reviewers were involved in this systematic review of evidence with discussions and decisions conducted online. The selected papers were systematically reviewed thematically, and their methodologies assessed by AR with independent verifications by TF who also ensured no relevant articles were missing in the systematic review. Then, eligibility of the full-text records following screening of abstracts was conducted by AR and further corroborated by DS and TF. The three authors were familiar with reviewing and presenting descriptive assessments of quantitative results which should minimize the potential bias arising from reporting conflicting results. Any differing judgements on the selection of articles and extraction of results were resolved with the expert verdict of DS who also gave the final confirmation on credibility of the synthesis with regards to the climatic variables’ influence on malaria dynamics. AR compiled the retrieved and summarized data of studies selected from the eligibility assessment stage into a separate review table in a shared Microsoft Word document (Version 2207 Build 16.0.15427.20182). DS and TF worked independently in corroborating the extracted data and narrative outlined in the shared review table. The systematic review flowchart to document our research process according to PRISMA conventions is shown in Figure 1.

### Data extraction

Data was extracted from the studies that met the eligibility conditions. The studies were imported from their respective online databases and managed with the reference management software Zotero (Version 6.0.13, RRID: SCR_013784). The information included in the extractions were title, author and year of publication, location of study, period of study, independent and dependent variables, the scale and measure of the variables, quantative analysis methods, and summary of results. These extracted data were retrieved and summarized for qualitative synthesis within a table in a shared Microsoft Word document that was independently corroborated by the reviewing authors.

### Data synthesis

The study utilizes a narrative synthesis method to describe the overarching influence of climatic variables indicated by meteorological measures such as temperature, humidity, wind speed and precipitation on malaria prevalence and/or incidence in countries across Southeast Asia. The strength and direction of the influence between variables is derived from the outcomes and summaries of the quantitative correlation, regression or mathematical models presented in the reviewed studies. Every climatic variable found throughout the review is described in the narrative synthesis irrespective of the number of studies that include them for the quantitative models. The reported outcomes of studies included in the final review are tabulated to group them according the the temperature, precipitation, humidity, and windspeed indicators as a meteorological proxy variables of climate change against their respective effects on malaria. Any incomplete reported outcomes in statistical tables found within the reviewed articles were thoroughly searched in the textual section of the articles with corroborations from the three authors. Categorized tabulations of the summarized articles were made using Microsoft Word’s table features and shared amongst the three authors concurrently.

## Results

During the first stage of the review process, a total of 535 records were identified from four different databases. 10 records were written in Indonesian and 525 in English. We then conducted a deduplication process which then left 391 records for abstract screening. After 391 of the articles were screened from their abstracts, 352 were excluded as they included studies outside the Southeast Asian region, were not ecological analyses or did not indicate the inclusion of more than one climatic variable. We were then left with 39 records for full-text eligibility assessment. In this final stage, 20 full-text articles were excluded as they did not include malaria cases as an outcome variable and did not use multi-year data for analysis in addition to the similar exclusion reasons of the previous stage. This left us with a total of 19 articles selected for the final review which we have summarized categorically and presented in Tables 1– 4. A meta-analysis attempt was made following the Hedges-Olin and Hunter-Schmidt method to assess sets of regression and correlation coefficients by way of pooled fixed effects. ^17^^–^^19^ However, due to insufficiencies in the reported statistical results and variations in methodologies in the 19 selected articles, a meta-analysis report could not be made in this study as we’ve detailed in our added underlying data. ^20^ This review is therefore only a narrative systematic review.

**Table 1.** Summary of published articles assessing the association between temperature with malaria cases in various locations throughout Southeast Asia.

Reviewed study, period and location	Variable measurement scale and method	Results summary (Temperature)
Purworejo, Indonesia [2005–2014] ^24^	Variable: Monthly maximum & minimum temperature with lags of one, two, three and 12. Method: Poisson regression, quasi-Poisson regression, and negative binomial regression.	The temperature variable used exhibited statistical significance in all models with the Poisson & quasi-Poisson regression against monthly positive malaria cases (p < 0.05, p < 0.01 and p < 0.001). Standard errors were not reported for these two models. After accounting for the dispersion parameter and AIC GoF test, the negative binomial model was selected as the best model. In the model, temperature variables did not maintain statistical significance.
Menoreh Hill, Indonesia [2005–2015] ^25^	Variable: Mean monthly temperature with lags of one, two, three and 12 Method: Poisson regression, quasi-Poisson regression, and negative binomial regression	The temperature variable used with lags exhibited statistical significance only in the Poisson regression model against monthly positive malaria cases (p < 0.05 and p < 0.001). At lags one and two, mean temperature negatively influences malaria cases, while at lags three and 12 the coefficient is positive. Standard errors were not reported for these two models. After accounting for the dispersion parameter and AIC GoF test, the negative binomial model was selected as the best model. In the model, temperature variables did not maintain statistical significance.
Bengkulu City, Indonesia [2011–2013] ^21^	Variable: Mean monthly temperature Method: Bivariate Pearson’s correlation	The correlation between the temperature variable used and monthly malaria cases did not exhibit statistical significance.
East Sumba Regency, Indonesia [2013–2019] ^22^	Variable: Mean yearly temperature Method: Bivariate Pearson’s correlation & linear regression	Correlation between the temperature variable used and malarial annual parasite index (API) did not show statistical significance at p < 0.05. In the linear regression model with yearly humidity and precipitation as added variables, statistical significance was reported for the temperature variable ( *β* = -8.803, R ^2^ = 0.879, p < 0.05).
Pandeglang Regency, Indonesia [2005–2010] ^23^	Variable: Mean monthly temperature Method: Bivariate Pearson’s correlation	Correlation between monthly temperature with monthly malaria cases is not statistically significant. The authors state that this is due to temperature data range in the regency averaging below the optimum temperatures for malaria vector reproduction.
Jayapura Regency, Indonesia [2011–2018] ^26^	Variable: Mean yearly temperature Method: Bivariate Spearman’s rank correlation	Correlation between yearly temperature with API is not statistically significant. The authors argue that as the research was limited to yearly data, a more granular monthly data is recommended for better analysis of the rank-correlation between climatic variables and malaria incidence.
Rajabasa & Padangcermin District, Lampung, Indonesia [2008–2009] ^27^	Variable: Mean monthly temperature Method: Bivariate Pearson’s correlation & linear regression	The authors of this study did not correlate temperature directly with malaria incidence. Correlation and linear regression results between the temperature variable used and malaria incidence via mosquito density (in man-biting rate/MBR) did not exhibit statistical significance.
Bangladesh, Cambodia, India, Indonesia, Laos, Malaysia, Myanmar, the Philippines, Thailand, and Vietnam [1980–2009] ^30^	Variable: Global Infectious Disease Epidemiology Online Network (GIDEON) malaria outbreak record Method: Generalized Additive Models (Logistic link function	Through spline-fitting, the authors found a non-linear relationship between maximum temperature and outbreak probability of mosquito-borne infectious diseases that is statistically significant (p = 0.0085). The findings suggest a parabolic peak of outbreak probability at about 33.5°C.
Kanchanaburi province, Thailand [1999–2005] ^36^	Variable: Mean monthly temperature Method: Regression analysis and information value analysis (IV)	In the regression analysis, temperature against monthly malaria incidence did not exhibit statistical significance to be used in the information value analysis to determine malaria risk levels.
Ayeyarwady region, Myanmar [2013–2017] ^35^	Variable: Mean annual temperature Method: Poisson regression	The temperature variable used did not exhibit statistical significance against annual malaria incidence per 1000, annual malaria testing, and annual malaria positivity rate.
77 provinces across Thailand [2012–2017] ^34^	Variable: weekly mean temperature Method: Bivariate Pearson’s correlation	The authors of this study did not indicate the degree of correlation between the independent and dependent variables. The study instead maps the provinces where statistical significances were found between the variable interactions. For mean temperature and malaria incidence rates, statistically significant correlation was found in 44 provinces (p < 0.05) with a concentration in the western and southern regions of Thailand.
Songkhla province, Thailand [1982–2018] ^33^	Variable: mean annual temperature Method: linear regression	The temperature variable used did not exhibit statistical significance against annual malaria morbidity rates per 1,000. The authors note that as the study site is a coastal area with saline peat swamps unfavorable for mosquito-borne diseases like dengue and malaria, lack of variations in the morbidity rates of the two diseases did not align with fluctuations in climatic variables across the province.
23 districts in Buriram province & 17 districts in Surin province, Thai-Cambodia border [2008–2012] ^38^	Variable: yearly mean, maximum & minimum temperatures Method: linear regression	The temperature variable used did not exhibit statistical significance against annual malaria morbidity rates.
19 provinces in Thailand [1994–2001] ^28^	Variable: mean monthly temperature Method: Neural network analysis	Neural network method was conducted to create a model for predicting malaria transmission based on independent variable data. The resulting model correctly predicted monthly malaria cases for 11 provinces and while overestimating for eight provinces in 2001.
Koh Chang, Thailand [2001–2011] ^29^	Variable: maximum, minimum and mean monthly temperature with lags of 0–7 months Method: Poisson regression & quasi-Poisson regression analysis with added cubic spline smoothing models	Maximum temperature, mean temperature, and minimum temperature exhibited statistical significance at p < 0.01. The first two are positively correlated with malaria cases at month 1 ( *r* = 0.150 & *r* = 0.190), while the latter shows inverse relationships at month 10 ( *r* = -0.233). With the added spline-fitted models, maximum temperatures ranging from 29–32.5°C at lags one, four and five positively explains malaria cases. Beyond that maximum temperature range however, the relationship reverses.
670 districts in Vietnam [2007–2008] ^31^	Variable: Spatial median temperature per month Method: Zero-inflated Poisson regression analysis	Spatial median temperatures were split into maximum and minimum temperatures. However, maximum temperature was excluded from the models due to collinearity with the minimum temperature. Temperature had varying effects across the districts in Vietnam. Minimum temperature showed statistically significant negative relationship with monthly plasmodium falciparum cases in the Northwest ( *β* = -0.24, 95% CrI = -0.32 to -0.17), Central Highlands ( *β* = -0.23, 95% CrI = -0.26 to -0.20) and Southeast regions ( *β* = -0.14, 95% CrI = -0.17 to -0.11) with the opposite relationships found elsewhere. Similar log relative risk relationships reported as *β-*coefficients are found with plasmodium vivax cases.
Phu Yen province, Vietnam [2005–2016] ^37^	Variable: Mean, minimum & maximum monthly temperatures Method: Spatial regression analysis, Poisson regression analysis & Zero-inflated Poisson regression	Only minimum temperature and mean precipitation variable was used in the regression model following model selection for best fit covariates. The final model suggests that for every 1°C reduction in minimum temperatures, *P. falciparum* and *P. vivax* cases decrease by 7.7% and 10.5% respectively (RR = 0.923, 95% CrI = 0.903 to 0.944 and RR = 0.895, 95% CrI = 0.874 to 0.917).
9 administrative districts in Tak province, Thailand [2012–2015] ^32^	Variable: Mean, minimum, maximum & median monthly temperatures with lags of one, two and three months Method: Bivariate Pearson’s correlation and linear regression	Statistically significant relationships were found for several temperature variables against total malaria incidence with detail as follows: • Median temperature at lag 1 ( *r* = 0.38, p = 0.008) • Median temperature at lag 2 ( *r* = 0.47, p = 0.001) • Mean temperature at lag 1 ( *r* = 0.38, p = 0.008) • Mean temperature at lag 2 ( *r* = 0.47, p = 0.001)
Vietnam [2011–2015] ^46^	Variable: Mean monthly temperature Method: Spatial autocorrelation & multilevel negative binomial regression analysis	The regression results of the study show that for every point increase in the temperature variable used, monthly malaria cases rise by 0.4% (95% CrI = 0.2 to 0.7, p < 0.05).

**Table 2.** Summary of published articles assessing the association between humidity with malaria cases in various locations throughout Southeast Asia.

Reviewed study, period and location	Variable measurement scale and method	Results summary (Humidity)
Purworejo, Indonesia [2005–2014] ^24^	Variable: Monthly maximum & minimum humidity with lags of one, two, three and 12. Method: Poisson regression, quasi-Poisson regression, and negative binomial regression.	The humidity variable used exhibited statistical significance in all models with the Poisson & quasi-Poisson regression against monthly positive malaria cases (p < 0.05, p < 0.01 and p < 0.001). Standard errors were not reported for these two models. After accounting for the dispersion parameter and AIC GoF test, the negative binomial model was selected as the best model. In the model, maximum humidity at lag 2 maintains statistical significance ( *β* = -0.110, SE = 0.023, p < 0.001).
Bengkulu City, Indonesia [2011–2013] ^21^	Variable: Mean monthly humidity Method: Bivariate Pearson’s correlation	The correlation between the humidity variable used and monthly malaria cases did not exhibit statistical significance.
East Sumba Regency, Indonesia [2013–2019] ^22^	Variable: Mean yearly humidity Method: Bivariate Pearson’s correlation & linear regression	Correlation and regression analysis between the humidity variable used and malarial annual parasite index (API) is not statistically significant.
Pandeglang Regency, Indonesia [2005–2010] ^23^	Variable: Mean monthly humidity Method: Bivariate Pearson’s correlation	Correlation between monthly humidity with monthly malaria cases is not statistically significant. The authors state that this is due to humidity data range in the regency averaging below the optimum temperatures for malaria vector reproduction.
Jayapura Regency, Indonesia [2011–2018] ^26^	Variable: Mean yearly humidity Method: Bivariate Spearman’s rank correlation	Correlation between yearly humidity with API is not statistically significant. The authors argue that as the research was limited to yearly data, a more granular monthly data is recommended for better analysis of the rank-correlation between climatic variables and malaria incidence.
Rajabasa & Padangcermin District, Lampung, Indonesia [2008–2009] ^27^	Variable: Mean monthly humidity Method: Bivariate Pearson’s correlation & linear regression	The authors of this study did not correlate humidity directly with malaria incidence. Correlation and linear regression results between the humidity variable used and malaria incidence via mosquito density (in man-biting rate/MBR) exhibited statistically significant results ( *r* = 0.636, p = 0.026)
Kanchanaburi province, Thailand [1999–2005] ^36^	Variable: Mean monthly humidity Method: Regression analysis and information value analysis (IV)	In the regression analysis, humidity against monthly malaria incidence did not exhibit statistical significance to be used in the information value analysis to determine malaria risk levels.
77 provinces across Thailand [2012–2017] ^34^	Variable: weekly mean relative humidity Method: Bivariate Spearman’s Rank correlation	The authors of this study did not indicate the degree of correlation between the independent and dependent variables. The study instead maps the provinces where statistical significances were found between the variable interactions. For mean relative humidity and malaria incidence rates, statistically significant correlation was found in 35 provinces (p < 0.05) with a concentration in the northern and northeastern regions of Thailand.
Songkhla province, Thailand [1982–2018] ^33^	Variable: mean annual relative humidity Method: linear regression	The humidity variable used did not exhibit statistical significance against annual malaria morbidity rates per 1,000. The authors note that as the study site is a coastal area with saline peat swamps unfavorable for mosquito-borne diseases like dengue and malaria, lack of variations in the morbidity rates of the two diseases did not align with fluctuations in climatic variables across the province.
23 districts in Buriram province & 17 districts in Surin province, Thai-Cambodia border [2008–2012] ^38^	Variable: annual relative humidity Method: linear regression	Despite indicating a linear regression approach, the authors only reported R-squared and adjusted R-squared values for the interaction between the variables. The annual relative humidity exhibited statistical significance against annual malaria morbidity rates (adjusted R ^2^ = 0.057, p = 0.03).
19 provinces in Thailand [1994–2001] ^28^	Variable: mean monthly relative humidity Method: Neural network analysis	Neural network method was conducted to create a model for predicting malaria transmission based on independent variable data. The resulting model correctly predicted monthly malaria cases for 11 provinces and while overestimating for eight provinces in 2001.
Koh Chang, Thailand [2001–2011] ^29^	Variable: mean monthly relative humidity with lags of 0–7 months Method: Poisson regression & quasi-Poisson regression analysis with added cubic spline smoothing models	Relative humidity shows statistical significance at p < 0.01 with correlation of *r =* -0.190 at month 10. The authors add that malaria cases associate positively with the preceding one- and two-month humidity levels but are reversed when associated with the levels of three months earlier.
9 administrative districts in Tak province, Thailand [2012–2015] ^32^	Variable: Mean, minimum, maximum & median monthly relative humidity with lags of one, two and three months Method: Bivariate Pearson’s correlation and linear regression	Statistically significant relationships were found for several relative humidity variables with detail as follows: • Mean relative humidity at lag two ( *r =* -0.35, p = 0.019) • Mean relative humidity at lag three ( *r =* -0.47, p = 0.001)
Vietnam [2011–2015] ^46^	Variable: Mean monthly humidity Method: Spatial autocorrelation & multilevel negative binomial regression analysis	The resulting regression coefficient for the mean humidity variable used did not exhibit statistical significance.

**Table 3.** Summary of published articles assessing the association between precipitation with malaria cases in various locations throughout Southeast Asia.

Reviewed study, period and location	Variable measurement scale and method	Results summary (Precipitation)
Purworejo, Indonesia [2005–2014] ^24^	Variable: Monthly precipitation with lags of one, two, three and 12. Method: Poisson regression, quasi-Poisson regression, and negative binomial regression.	The precipitation variable used exhibited statistical significance in all models with the Poisson & quasi-Poisson regression against monthly positive malaria cases (p < 0.05, p < 0.01 and p < 0.001). Standard errors were not reported for these two models. After accounting for the dispersion parameter and AIC GoF test, the negative binomial model was selected as the best model. In the model, the following precipitation variables maintained statistical significance: • Precipitation at lag 3 ( *β* = 0.0008, SE = 0.0003, p < 0.05). • Precipitation at lag 12 ( *β* = 0.0009, SE = 0.0003, p < 0.05).
Menoreh Hill, Indonesia [2005–2015] ^25^	Variable: Mean monthly precipitation with lags of one, two, three and 12 Method: Poisson regression, quasi-Poisson regression, and negative binomial regression	The precipitation variable used with lags exhibited statistical significance only in the Poisson regression model against monthly positive malaria cases (p < 0.05, p < 0.01 and p < 0.001). After accounting for the dispersion parameter and AIC GoF test, the negative binomial model was selected as the best model. In the model, temperature variables did not maintain statistical significance.
Bengkulu City, Indonesia [2011–2013] ^21^	Variable: Mean monthly precipitation Method: Bivariate Pearson’s correlation	The correlation between the precipitation variable used and monthly malaria cases exhibit statistical significance ( *r* = -0.431, p = 0.009). The author also used number of rainy days which showed a negative correlation ( *r* = -0.349, p = 0.037).
East Sumba Regency, Indonesia [2013–2019] ^22^	Variable: Mean yearly precipitation Method: Bivariate Pearson’s correlation & linear regression	The correlation between the precipitation variable used and yearly API exhibited statistical significance ( *r* = 0.787, p = 0.036). From the linear regression model, a 1 mm increase precipitation is associated with a 0.028 increase in API ( *β* = 0.028, R ^2^ = 0.879, p < 0.05).
Pandeglang Regency, Indonesia [2005–2010] ^23^	Variable: Mean monthly precipitation Method: Bivariate Pearson’s correlation	Correlation between monthly precipitation with monthly malaria cases is not statistically significant.
Jayapura Regency, Indonesia [2011–2018] ^26^	Variable: Mean yearly precipitation Method: Bivariate Spearman’s rank correlation	Correlation between yearly precipitation with API is not statistically significant. The authors argue that as the research was limited to yearly data, a more granular monthly data is recommended for better analysis of the rank-correlation between climatic variables and malaria incidence.
Rajabasa & Padangcermin District, Lampung, Indonesia [2008–2009] ^27^	Variable: monthly precipitation index Method: Bivariate Pearson’s correlation & linear regression	The authors of this study did not correlate temperature directly with malaria incidence. Results indicate a positive correlation between precipitation index with MBR ( *r* = 0.754, p = 0.005). The model with precipitation index as a predictor resulted in an R-squared value that shows 56.9% of the variations in Anopheles density is explained by variations in the precipitation index.
Bangladesh, Cambodia, India, Indonesia, Laos, Malaysia, Myanmar, the Philippines, Thailand, and Vietnam [1980–2009] ^30^	Variable: Global Infectious Disease Epidemiology Online Network (GIDEON) malaria outbreak record Method: Generalized Additive Models (Logistic link function)	In the spline-fitting model, maximum precipitation’s non-linear relationship with malaria outbreak exhibited no statistical significance. The authors argue that the use of maximum precipitation is insignificant as yearly precipitation trends do not explain mosquito-borne diseases because decreased precipitation could actually increase outbreak risk whilst more rainfall would do the opposite.
Kanchanaburi province, Thailand [1999–2005] ^36^	Variable: Mean monthly rainfall Method: Regression analysis and information value analysis (IV)	In the regression analysis, rainfall against monthly malaria incidence showed statistical significance to be used in the information value analysis to determine malaria risk levels. Results of the IV analysis shows that moderate risk areas are those with average monthly rainfall levels of 1500–2000 mm over wetlands, bare lands, and water bodies at elevation levels of 300–500 m. Meanwhile, high risk areas have average monthly rainfall levels of 400–900 mm and 900–1500 mm over land types such as agricultural area, forest area and urban areas with elevations of 100–300 m.
Ayeyarwady region, Myanmar [2013–2017] ^35^	Variable: Annual temperature Method: Poisson regression	The temperature variable used did not exhibit statistical significance against annual malaria incidence per 1000, annual malaria testing, and annual malaria positivity rate.
77 provinces across Thailand [2012–2017] ^34^	Variable: weekly mean rainfall Method: Bivariate Pearson’s correlation	The authors of this study did not indicate the degree of correlation between the independent and dependent variables. The study instead maps the provinces where statistical significances were found between the variable interactions. For mean rainfall and malaria incidence rates, statistically significant correlation was found in 38 provinces (p < 0.05) with a concentration in the northern and western regions of Thailand.
Songkhla province, Thailand [1982–2018] ^33^	Variable: mean annual rainfall Method: linear regression	The rainfall variable used did not exhibit statistical significance against annual malaria morbidity rates per 1,000. The authors note that as the study site is a coastal area with saline peat swamps unfavorable for mosquito-borne diseases like dengue and malaria, lack of variations in the morbidity rates of the two diseases did not align with fluctuations in climatic variables across the province.
23 districts in Buriram province & 17 districts in Surin province, Thai-Cambodia border [2008–2012] ^38^	Variable: yearly mean, maximum & minimum temperatures Method: linear regression	Despite indicating a linear regression approach, the authors only reported R-squared and adjusted R-squared values for the interaction between the variables. The precipitation variable used did not exhibit statistical significance against annual malaria morbidity rates.
19 provinces in Thailand [1994–2001] ^28^	Variable: monthly precipitation amount and precipitation amount in the previous month Method: Neural network analysis	Neural network method was conducted to create a model for predicting malaria transmission based on independent variable data. The resulting model correctly predicted monthly malaria cases for 11 provinces and while overestimating for eight provinces in 2001.
Koh Chang, Thailand [2001–2011] ^29^	Variable: mean monthly rainfall with lags of 0–7 months Method: Poisson regression & quasi-Poisson regression analysis with added cubic spline smoothing models	Throughout the study period, most malaria cases are reported during months with lesser rainfall. Evidently, the statistically significant (p < 0.01) highest correlation between average rainfall and malaria was *r =* -0.183 at month 9. With the added spline-fitted models, rainfall ranges of 0 to 500 mm show a positive relationship with malaria cases by a lag of two and five months. As precipitation levels go above 500 mm, malaria cases exhibit a decline.
670 districts in Vietnam [2007–2008] ^31^	Variable: Spatial median precipitation per month Method: Zero-inflated Poisson regression analysis	The precipitation variable used had varying effects across the districts in Vietnam. Precipitation showed statistically significant positive relationship (α ≤ 0.05) with monthly plasmodium falciparum and plasmodium vivax cases in the Northwest, North Central Coast, South Central Coast and Central Highland regions. This relationship is inversed in the Southeast and Mekong Delta regions.
Phu Yen province, Vietnam [2005–2016] ^37^	Variable: Mean monthly precipitation Method: Spatial regression analysis, Poisson regression analysis & Zero-inflated Poisson regression	Only minimum temperature and mean precipitation variable was used in the regression model following model selection for best fit covariates. The final model suggests that for every 10 mm increase in precipitation is associated with a 5.4% and 3.2 % increase in *P. falciparum* and *P. vivax* cases respectively (RR = 1.054, 95% CrI = 1.051 to 1.057 and RR = 1.032, 95% CrI = 1.029 to 1.035).
9 administrative districts in Tak province, Thailand [2012–2015] ^32^	Variable: Monthly total rainfall with lags of one, two and three months Method: Bivariate Pearson’s correlation and linear regression	The precipitation variable used did not exhibit statistical significance against monthly malaria cases.
Vietnam [2011–2015] ^46^	Variable: monthly cumulative rainfall Method: Spatial autocorrelation & multilevel negative binomial regression analysis	The regression results of the study shows that every additional millimeter of precipitation leads to a 2.1% reduction in malaria cases (95% CrI = -2.3 to -1.9, p < 0.05).

**Table 4.** Summary of published articles assessing the association between windspeed with malaria cases in various locations throughout Southeast Asia.

Reviewed study, period and location	Variable measurement scale and method	Results summary (Windspeed)
Bengkulu City, Indonesia [2011–2013] ^21^	Variable: Mean monthly windspeed Method: Bivariate Pearson’s correlation	The correlation between the windspeed variable used and monthly malaria cases did not exhibit statistical significance.
Jayapura Regency, Indonesia [2011–2018] ^26^	Variable: Mean yearly temperature Method: Bivariate Spearman’s rank correlation	Correlation between yearly windspeed with API is not statistically significant. The authors argue that as the research was limited to yearly data, a more granular monthly data is recommended for better analysis of the rank-correlation between climatic variables and malaria incidence.

## Discussion

### Temperature and malaria (19 studies)

Temperatures across different locations in Southeast Asia proved to be a worthy inclusion for analyzing the relationship with malaria cases based on the reviewed studies. Both *Anopheles* mosquitoes as the vector for malaria and the parasitic agent, Plasmodium group, rely on optimally warm temperatures. ^21^^–^^27^ The optimal temperature for Anopheline reproduction according to Mau *et al*. (2020) is between 25–27°C whilst the Plasmodium group’s extrinsic cycle is optimum within the 20–30°C range. ^22^ This implies that as temperatures become warmer within the optimum range, the duration of incubation is shortened, and mosquitoes become infective much sooner. ^22^^,^^24^^,^^26^

That said, the quantitative analyses conducted in the reviewed studies exhibited varying results with temperature variables being averaged over a year, a month, split into maximum and minimum ranges as well as added lagged variations. For instance, in Kiang *et al*.'s (2006) neural network analysis, the composite use of mean monthly temperature with other climatic variables and vegetation index resulted in a model configuration to assess malaria cases with a training accuracy of 73% and testing accuracy of 53%. The model was developed based on variable data from 19 provinces across Thailand throughout a seven-year period (1994–2001).

Meanwhile, a study of the Koh Chang district in Thailand throughout 2001–2011 indicates that maximum temperature and mean temperature are positively correlated with malaria cases ( *r* = 0.150 and *r* = 0.190 at α ≤ 0.01) by one year according to the count regression models. ^29^ Similarly, Rejeki *et al*. (2018) also utilized count models in which maximum and minimum temperatures were included with the addition of lagging by one, two, three and 12 months. The results of their baseline Poisson model suggest that minimum and maximum temperatures have a significant influence on monthly malaria cases at the negative and positive directions respectively. However, after the inclusion of a dispersion parameter and testing for fit, a negative binomial model was selected in Rejeki *et al*. (2018) in which maximum and minimum temperatures exhibited no significance at α ≤ 0.05.

In Mau *et al*. (2020), temperature was also shown to have a significant influence on malaria cases in their linear regression model. The difference being those temperatures were averaged over a year and negatively influenced malaria cases, which were measured as annual parasite incidence (API). The results in Rejeki *et al*. (2018) and Mau *et al*. (2020) suggest that further increases of temperatures beyond certain levels will be associated with reductions in malaria cases – in line with the optimum range for both Anopheline reproduction and Plasmodia incubation. In the Rejeki *et al*. (2018) Purworejo study, maximum temperatures recorded in the study period ranged between 28–30°C, ^24^ while the average temperatures between 2013–2019 at the study site in Mau *et al*. (2020) ranged between 25.13–27.58°C (which is already the optimum levels for Anopheline reproduction). Interestingly, the studies of Ninphanomchai *et al*. (2014) and Servadio *et al*. (2018) have offered their explanatory evidence through a non-linear thresholding effect with spline-fitted models that establish a peak positive effect of temperature at around 30°C. ^29^^,^^30^

Moreover, another alternative take on the temperature-malaria case relation is found in studies by Bui *et al*. (2011), Mercado *et al*. (2019) and Noppradit *et al*. (2021) who similarly argue that other factors confound the role temperature plays in variations of malaria cases. In their study of 670 districts across Vietnam between 2007–2008, Bui *et al*. (2011) found that temperature variables have district specific effects with many regions exhibiting opposite interactions with malaria cases, i.e., in some regions malaria cases soar as temperatures rise while others reduce with increased heat. The same is stated in Kotepui and Kotepui (2018) and Noppradit *et al*. (2021), where the latter argues that the lack of statistical significance between malaria and temperature variables in their study was due to topographic factors of the study site which were unfavorable to malaria from the commencement of the study.

### Precipitation and malaria (19 studies)

The next climatic variable analyzed for its association with malaria in the reviewed studies was precipitation. Although the amount of rain does not directly affect both vector and parasite proliferation, low to medium intensity rain creates reservoirs in the form of pools and puddles for *Anopheles* to breed. ^21^^–^^27^ Aside from forming breeding sites, precipitation also leads to increased relative humidity which prolongs the age of infective adult *Anopheles* mosquitoes as explained in the following section. ^22^^,^^24^^–^^26^

Across the 19 reviewed studies, the measures of precipitation as an independent variable varies. A mean yearly precipitation is used in Sandy & Wike (2019) and Mau *et al*. (2020) for their study in Indonesia, Gallalee Sarah *et al*. (2021) in Myanmar, and Noppradit *et al*. (2021) in Thailand while mean monthly precipitation is used in Kiang *et al*. (2006), Jeefoo *et al*. (2009), Bui *et al*. (2011), Ninphanomchai *et al*. (2014), Nurmala (2017), Rejeki *et al*. (2018), Rejeki *et al*. (2019), Mercado *et al*. (2019), and Wangdi *et al*. (2020). Similar to the latter articles, Jubaidi (2015) also includes mean monthly precipitation in addition to the number of rainy days per month. Suwito *et al*. (2010) on the other hand uses a precipitation index. They defined the index as the product of the sum of precipitation and number of rainy days within a given month that is then divided by the total number of days in said month. ^27^

The relationship between precipitation between malaria cases across the reviewed studies was inconclusive as the resultsof some studies did not exhibit statistical significance for the variable. ^30^^,^^32^^,^^33^^,^^35^^,^^38^ The study by Suwito *et al*. (2010) did not statistically associate precipitation index with malaria cases, but their resulting correlation between the former with *Anopheles* density measured by man-biting rate (MBR) exhibited a positive relationship that was statistically significant. As for direct associations with statistical significance, the correlation and linear regression results in Mau *et al*. (2020) showed that yearly precipitation has a positively linear relationship with API where their linear regression model implies that for every 1 mm increase in precipitation, API cases increase by 0.028. The negative binomial regression results in Rejeki *et al*. (2018) also indicate that a 1 mm increase in precipitation has a positive influence on malaria cases by 0.08% and 0.09% with the use of three-month and 12-month lags respectively. However, in contrast to the three previous studies, results in Jubaidi (2015) showcases negative correlations that are statistically significant between monthly precipitation and number of rainy days with monthly malaria incidence ( *r* = -0.431, α ≤ 0.01 and *r* = -0.349, α ≤ 0.05 respectively). Taking into account the previously mentioned studies, the results in Jubaidi (2015) indicate the need to take precipitation as a lagging indicator for direct associations with malaria cases as suggested in Kim *et al.* (2012), Krefis *et al.* (2011), and Wu *et al.* (2017). ^39^^–^^41^

### Humidity and malaria (14 studies)

As previously mentioned, humidity as a meteorological measure of climate is an important indicator for malaria cases as it enables the lifespan of an infective adult *Anopheles* mosquito – where relative humidities of at least 60% and above optimize the mosquitoes’ activity to bite and infect. ^23^^,^^24^^,^^26^^,^^27^

Only seven of the reviewed studies had results where humidity was a statistically significant independent variable. That said, the results were inconclusive. In the negative binomial model of Rejekti *et al*. (2018), results suggest that a 1% increase in maximum relative humidity is associated with a 10.47% decrease in malaria cases after two months. However, the results of Suwito *et al*. (2010) instead further validate the influence of humidity on *Anopheles* activity to bite and infect as average humidity is shown to have a statistically significant positive correlation with MBR. Narrative background for humidity posed by the other reviewed studies, where previous prevailing studies are also referred to, would suggest the latter study as being the sounder evidence for humidity’s relationship with malaria cases (albeit indirectly). The authors in the former study unfortunately did not provide any theoretical explanations as to why their two-month lagged maximum humidity was associated with a decrease in malaria cases. However, an argument could be made regarding the range of the maximum humidity throughout the period and location of study. In Purworejo, the maximum humidity between 2005–2014 varied between 83–99%, which is well beyond the 60% necessary optimum for *Anopheles* mosquito activity. Alternatively, the results in Rejeki *et al*. (2018) could suggest that the use of humidity as a lagged indicator associated with malaria cases is unwarranted.

### Windspeed and malaria (Two studies)

Only two of the reviewed articles included windspeed as a climatic variable that was assessed for its relationship with malaria cases. ^21^^,^^26^ Based on references included in their article, Sandy and Wike (2019) state that windspeed has an influence on *Anopheles* mosquitoes’ flight range, hence enabling an expanded scope of humans to bite. That said, the evidence in both Jubaidi (2015) and Sandy and Wike (2019) indicate that the resulting correlation between windspeed with monthly malaria incidence and API respectively did not exhibit statistical significance. The authors in both attribute the narrow windspeed range across the periods and their respective locations of study as the potential reason for the variable not resulting in statistically significant correlations.

## Conclusions

Following previous systematic reviews of evidence on changing climatic variables’ relationship with malaria for a given region such as Babaie *et al.* (2018), ^15^ Fischer *et al.* (2020), ^16^ and Bai *et al.* (2013), ^42^ this review finds that changing temperatures, precipitation and humidity across a multi-year period are correlated with malaria cases in various regions throughout Southeast Asia. The established evidence, however, was only limited to 19 articles with most studies in Indonesia (7), Vietnam (3) and Thailand (7). Many other studies were also excluded from this review as they either utilized only a single meteorological measure, which undercuts the complex dynamics of climatic variables or claimed to assess changing climatic variables despite only analyzing a single-year period, which is not informative for exhibiting a change of climate normals that is indicated by multi-year averages.

However, the exhibited evidence for the case of Southeast Asia suggests that further explorations could still be made with regards to the intricate dynamics of changing climatic variables with malaria incidence and/or prevalence across the region. Future research could incorporate added interactons with better inclusion of spatially varying confounders like distance from *Anopheles* reservoirs as done by Hasyim *et al*. (2018) ^43^ or changing land-use data through proxies such as the Normalized Difference Vegetation Index (NDVI) as done by Lubinda *et al*. ^44^ This is in addition to the suggested inclusion of non-climatic confounders such as availability of malaria interventions and programs, regionally specific topographic factors as well as behavioral and sociodemographic variables. ^23^^,^^24^^,^^28^^,^^31^^–^^33^^,^^38^

In conclusion, the findings of this systematic review of evidence could serve to inform the environmental ministries and health ministries of the respective Southeast Asian countries for climate change adaptation and malaria elimination strategies amidst climatic exacerbations.

## Data availability

### Underlying data

Figshare: Underlying data for ‘The relationship between climate change and malaria in South-East Asia: A systematic review of the evidence’. https://doi.org/10.6084/m9.figshare.20697298.v1. ^20^

### Reporting guidelines

Figshare: PRISMA checklist for ‘The relationship between climate change and malaria in South-East Asia: A systematic review of the evidence’. https://doi.org/10.6084/m9.figshare.20489235.v1. ^13^

Data are available under the terms of the Creative Commons Attribution 4.0 International license (CC-BY 4.0).
