# Supplementary material for: The relationship between climate change and malaria in South-East Asia: A systematic review of the evidence
Source: F1000Res. 2023 Jul 21;11:1555. Originally published 2022 Dec 22. [Version 2] doi: 10.12688/f1000research.125294.2 (PMC10585202; doi:10.12688/f1000research.125294.2)
Supplement: Peer review history

# Reviewer report: Reviewer response for version 2

**DOI:** 10.5256/f1000research.153273.r189413

**Published:** 2023-10-18

**Authors:** Imbahale S. Susan

**Affiliations:** 1 Technical University of Kenya, Nairobi, Nairobi County, Kenya

I have read the author's responses and I wish to confirm that I have no further concerns or corrections, please proceed with the indexing.

Are the rationale for, and objectives of, the Systematic Review clearly stated?

Yes

Is the statistical analysis and its interpretation appropriate?

Partly

Are sufficient details of the methods and analysis provided to allow replication by others?

Yes

Are the conclusions drawn adequately supported by the results presented in the review?

Partly

Reviewer Expertise:

NA

I confirm that I have read this submission and believe that I have an appropriate level of expertise to confirm that it is of an acceptable scientific standard.

# Reviewer report: Reviewer response for version 1

**DOI:** 10.5256/f1000research.137586.r181886

**Published:** 2023-07-14

**Authors:** Imbahale S. Susan

**Affiliations:** 1 Technical University of Kenya, Nairobi, Nairobi County, Kenya

**The relationship between climate change and malaria in South-East**

**Asia: A systematic review of the evidence**

 Congratulations to the authors for taking time to compile existing evidence on climate variability and malaria transmission in South East Asia (SEA) region. The authors need to consider providing enough background information to inform the reader on how similar or different the countries are in relation to climatic variables and malaria i.e. trends over time, so that its clear to the reader that all countries are similar. The changes in weather conditions do not operate in isolation, therefore this being a review, I suggest you consider including all other variables that might have contributed to the changes in malaria transmission in the region, so that you provide an overall picture of what exactly is going on.

**Here are some comments and questions for your consideration.**

  

1. The authors need to define what the term “climate” really refers to in their study.
  I would suggest the term “climate variability” rather than climate change in the title. I realize it’s the variations in the climatic variables that is leading to the changes in malaria transmission.
2. SEA region comprises of 10 countries, but the review focuses on 19 studies that only cover 3 countries on the SEA region. My concern is are the 3 countries a good representation of the whole region? If yes, where is the evidence suggesting that indeed the 3 represents the whole region with respect to the subject matter i.e. climate?
3. In view of the same concerns raised above. Malaria transmission and mosquito vectors are highly dependent on the local conditions, which in turn lead to modifications of the local weather conditions that ultimately translate to variations in climatic variables,
  a) There is no mention on the specific vectors responsible for malaria transmission in the 10 countries
  b) What are their climatic requirements in in terms of malaria transmission?
  c) Are the conditions for malaria transmission similar in all the 10 countries?
  d) Even within the same country, transmission is never uniform e.g. northern part of Indonesia might be different from southern part with respect to malaria. Local conditions, human activities are the other main drivers. This information is missing, and yet its what makes malaria transmission completely heterogenous.
4. The reader needs to be informed of any assumptions that the authors might have put into consideration.
5. The concerns raised should also inform you on the recommendations for future studies that are looking at climate variability and its effects on vector borne disease transmission.
6. Consider revisiting the conclusion of the study and state clearly, how helpful climate data would benefit the environment and health ministries?  And what are the specific recommendations to the SEA countries in terms of climate adaptation and malaria elimination?

Are the rationale for, and objectives of, the Systematic Review clearly stated?

Yes

Is the statistical analysis and its interpretation appropriate?

Partly

Are sufficient details of the methods and analysis provided to allow replication by others?

Yes

Are the conclusions drawn adequately supported by the results presented in the review?

Partly

Reviewer Expertise:

Medical entomologist interested in malaria vector ecology, control and public health in general

I confirm that I have read this submission and believe that I have an appropriate level of expertise to confirm that it is of an acceptable scientific standard, however I have significant reservations, as outlined above.

# Reviewer report: Reviewer response for version 1

**DOI:** 10.5256/f1000research.137586.r176897

**Published:** 2023-07-10

**Authors:** Eleanore Sternberg

**Affiliations:** 1 Tropical Health, London, England, UK

- I would simplify the writing and refocus the introduction on malaria. A brief history of the world’s climate is less relevant than covering some of the current literature on climate and malaria (and other vector-borne diseases). There is a substantial body of literature focused on climate change and malaria in Africa that would be relevant, plus modelling results on climate change and infectious disease.
- “This implies that as temperatures become warmer within the optimum range, the duration of incubation is shortened, and mosquitoes become infective much sooner” in the discussion is an important point. Once the temperature exceeds the optimal temperature (as it will undoubtedly in some places), that will slow or stop plasmodium development. This could potentially explain why increasing temperature is associated with more malaria in some instances and not in others.
- The results and discussion focuses on significant associations between malaria and climate variables, but the key result would be change over time. Change over time is mentioned in the conclusion, but I don’t think it’s clear in the way that results are presented whether the papers that are discussed showed temporal change as well as associations between malaria and climate indicators.
- Also related to change over time, studies that cover a long time period (e.g. reference 30 covers 1982 – 2018) are going to be more informative than studies that only cover a few years (e.g reference 18 that covers 2011-2013). The authors could consider highlighting the papers with long study periods in some way, for example by presenting them first or going into a bit more detail into their results. 

Are the rationale for, and objectives of, the Systematic Review clearly stated?

Partly

Is the statistical analysis and its interpretation appropriate?

Not applicable

Are sufficient details of the methods and analysis provided to allow replication by others?

Yes

Are the conclusions drawn adequately supported by the results presented in the review?

Partly

Reviewer Expertise:

Vector-borne diseases

I confirm that I have read this submission and believe that I have an appropriate level of expertise to confirm that it is of an acceptable scientific standard, however I have significant reservations, as outlined above.

# Reviewer report: Reviewer response for version 1

**DOI:** 10.5256/f1000research.137586.r179112

**Published:** 2023-07-10

**Authors:** Srinivasa Rao Mutheneni

**Affiliations:** 1 CSIR-Indian Institute of Chemical Technology, Hyderabad, India

Rahmani AA and colleagues present an interesting narrative systematic review of the relationship between climatic factors and malaria cases in the South-East Asia region. In their review analysis authors suggest that changing temperatures, precipitation and humidity across a multi-year period are correlated with malaria cases in various regions throughout Southeast Asia. However, I have some minor concerns about the framing and interpretation of the result. I suggest that authors carefully consider this and include the suggestions in the revision.

1. The need for the present review or the rationale for conducting the review is not clearly explained.
2. Though the inclusion and exclusion criteria are well defined, is the search strategy is comprehensive and transparent?
3. Is there any assessment of the risk of bias in the included studies?
4. It would have been better if the authors have included the recent and up-to-date literature. All the papers cited in the review used only the statistical methods but there is recent literature which has used machine learning and deep learning models to assess the relationship between climatic factors and malaria cases.
5. Please mention *Plasmodium* in italic throughout the manuscript.
6. In the result section, the number of records written in English is misquoted as 425, please change it to 525.
7. The review considered the studies conducted only in 3 countries and does not provide a balanced view of the topic.
8. Though numerous climatic variables influence malaria transmission, the study considered only temperature, precipitation, humidity and wind speed. The inclusion of the majority of the climate variables would provide a better insight into the situation.
9. Since all the climatic parameters exhibited variation in terms of significance in different regions, the discussion can be improved by mentioning major contributing factors in the specific region.
10. Please mention the consequences of the findings in the conclusion section in the context of climate change and malaria?

Are the rationale for, and objectives of, the Systematic Review clearly stated?

Yes

Is the statistical analysis and its interpretation appropriate?

Yes

Are sufficient details of the methods and analysis provided to allow replication by others?

Yes

Are the conclusions drawn adequately supported by the results presented in the review?

Yes

Reviewer Expertise:

Epidemiology, Climate Change and Public Health, Vector-Borne Diseases, Computational Biology, and Data Analytics.

I confirm that I have read this submission and believe that I have an appropriate level of expertise to confirm that it is of an acceptable scientific standard, however I have significant reservations, as outlined above.
